# Ten Years of Research on Automatic Voice and Speech Analysis of People With Alzheimer's Disease and Mild Cognitive Impairment: A Systematic Review Article

**DOI:** 10.3389/fpsyg.2021.620251

**Published:** 2021-03-23

**Authors:** Israel Martínez-Nicolás, Thide E. Llorente, Francisco Martínez-Sánchez, Juan José G. Meilán

**Affiliations:** ^1^Faculty of Psychology, University of Salamanca, Salamanca, Spain; ^2^Institute of Neuroscience of Castilla y León, University of Salamanca, Salamanca, Spain; ^3^Faculty of Psychology, University of Murcia, Murcia, Spain

**Keywords:** Alzheimer's disease, mild cognitive impairment, speech analysis, language impairment, speech impairment

## Abstract

**Background:** The field of voice and speech analysis has become increasingly popular over the last 10 years, and articles on its use in detecting neurodegenerative diseases have proliferated. Many studies have identified characteristic speech features that can be used to draw an accurate distinction between healthy aging among older people and those with mild cognitive impairment and Alzheimer's disease. Speech analysis has been singled out as a cost-effective and reliable method for detecting the presence of both conditions. In this research, a systematic review was conducted to determine these features and their diagnostic accuracy.

**Methods:** Peer-reviewed literature was located across multiple databases, involving studies that apply new procedures of automatic speech analysis to collect behavioral evidence of linguistic impairments along with their diagnostic accuracy on Alzheimer's disease and mild cognitive impairment. The risk of bias was assessed by using JBI and QUADAS-2 checklists.

**Results:** Thirty-five papers met the inclusion criteria; of these, 11 were descriptive studies that either identified voice features or explored their cognitive correlates, and the rest were diagnostic studies. Overall, the studies were of good quality and presented solid evidence of the usefulness of this technique. The distinctive acoustic and rhythmic features found are gathered. Most studies record a diagnostic accuracy over 88% for Alzheimer's and 80% for mild cognitive impairment.

**Conclusion:** Automatic speech analysis is a promising tool for diagnosing mild cognitive impairment and Alzheimer's disease. The reported features seem to be indicators of the cognitive changes in older people. The specific features and the cognitive changes involved could be the subject of further research.

## Introduction

Alzheimer's disease (AD) is a major neurocognitive disorder defined by a cognitive impairment that may interfere with independence. Memory deficit is the earliest and main symptom of the onset of AD, and it is accompanied by other cognitive deficits such as aphasia, apraxia, agnosia, and executive dysfunction. Despite memory deficit being the earliest and most characteristic symptom, there is also a growing interest in language impairments. This is attributable to the fact that language deficits are present as from the early—and even prodromal—stages (Cuetos et al., 2007), and they are therefore a key for early diagnosis. The challenge today is to accurately differentiate between patients with mild cognitive impairment (MCI) and those in the early stage of AD, on the one hand, and people with healthy aging, on the other. Hence, language becomes an important instrument to distinguish those impairments that might be concealing the prodromal stage of a neurodegenerative disease from other entities with a different involvement of language processes.

In the past few decades, major progress has been made in the development of biomarkers for AD diagnosis, such as Aβ and phosphorylated tau (Lee et al., 2019), neuroimaging techniques, and neuropsychological tests. However, these methods are limited by their high cost and invasive nature. Besides, no single biomarker can, by itself, accurately diagnose AD. Distinguishing the early stages of AD from the cognitive impairment associated with normal aging is a challenge, as a significant part of AD patients are asymptomatic during the preclinical stages of the pathological process, which is believed to last ~17 years (Villemagne et al., 2013; Jansen et al., 2015) until it compromises the person's cognition. During the long asymptomatic preclinical phase—and the progressive pathological changes that take places beneath the surface—(Jack et al., 2010), the speech and voice parameters associated with cognitive functions may anticipate the clinical manifestations of dementia and may be helpful in the early diagnosis of AD and in the development and assessment of preventive and therapeutic strategies.

Studies on language in dementia have tended to focus on what the patient says, rather than how they say it, thus ignoring changes in automatic language processes—e.g., voice—over the course of the disease. This is surprising because numerous studies have identified acoustic measures that highly correlate with pathological voice features or voice alterations (Markaki and Stylianou, 2011; Poellabauer et al., 2015), and voice and speech analysis (e.g., vocal phonation) are common diagnostic instruments used by some specialists, such as audiologists or speech pathologists.

Voice and speech are not only altered by disease but also change over a lifetime because of the normal aging processes. The first studies on the efficacy of articulatory control during speech movements in older people concluded that their performance is worse than that of young people. They suggested that movement amplitude accuracy tends to decline during aging, thereby having an impact on temporal voice parameters (Ballard et al., 2001). More recent studies have focused on a characteristic cluster of clinical features that appear in elderly people's voices, known as presbyphonia. This is an alteration in the voice, due to the aging process, caused by anatomical and physiological changes in the larynx and vocal tract of older people, and by the difficulties arising in the control of acoustic parameters (Alonso et al., 2001), and other mechanical, structural, and hormonal factors (Bruzzi et al., 2017). These changes explain the characteristic dysphonia among older people: a reduction in vocal range, a decrease in fundamental frequency (F0) in female voices (from normal levels around 248–175 Hz), and an increase in male voices (from 110 to 135–160 Hz). Additionally, a higher variation in frequencies (jitter) and amplitude in decibels (shimmer) appears, resonance is reduced, and there are more speech pauses (Linville, 2004).

These acoustic measures are parameters that cannot be naturally perceived by the human ear, as it perceives voice as a whole (sound, speech, and language), which is useful for communication but prevents us from distinguishing every component of voice. Fortunately, technological advances in the field of automatic speech analysis have enabled the objective extraction of these parameters using a method based on the Source-Filter Theory (Lieberman and Blumstein, 1988). According to this theory, the acoustic signal (generated in the vocal cords and composed of frequencies and harmonics) is shaped in the vocal tract through prosody into a temporally organized sequence characterized by formants or phonetically recognizable acoustic cues along with vocal noise. This technique focuses on the acoustic analysis of formants and pitch (monitoring the fundamental frequency over time) that are perceived as perceptual features by the auditory system and processed as prosodic suprasegmental features of speech (intonation, rhythm, and stress).

Prosody is one of the parameters most commonly studied in people with neurodegenerative diseases, as it refers to the phonetic and phonological properties of speech due to word choices, and to the rhythm and emphasis that reflect the speaker's attitude and emotional state (Ladd, 2008; Wagner and Watson, 2010). Prosody (specifically the analysis of speech rhythms—i.e., pauses, accents, or speech rate), along with other parameters related to temporal and acoustic voice measures, such as articulatory rhythm, voice intensity (analysis of sonority and variations in amplitude over time), emission time, and frequencies (variations in the frequencies of the sound signal, timbre, or formant structure), are used to discriminate between clinically defined groups (Gabani et al., 2009).

Regarding AD, forerunners such as Singh et al. (2001) have used an oral reading task to conclude that verbal rate, the mean duration of pauses, and standardized phonation time accurately discriminate between healthy older people and those with AD. Studies of this nature, with manually extracted parameters, have laid the foundations for automatic speech analysis. In 2006, Salhi and Cherif (2006) developed a new speech processing software and used it to analyze audios recorded by people with different pathologies. One of them belonged to an AD patient whose voice was distorted in formants F2 and F3. This finding paved the way for several studies on the analysis of voice in AD. Since this first analysis of the voice of an AD patient, studies exploring the behavioral consequences in vocal execution due to subtle changes in language processes have been gaining popularity. As of 2010, when the first study that directly addressed the subject was published, there have been a growing number of studies that seek to identify the changes in the voice of the elderly affected by AD, and, more recently, MCI. Most of these studies focus either on identifying characteristic voice parameters or use them to discriminate between healthy older people and those affected by the disease. However, the results are heterogeneous due to the variety of methods and voice features. Voice analysis programs extract dozens of parameters, many of them interrelated, but no clear picture is forthcoming of the speech aspects involved. Although the altered parameters found are attributed to cognitive changes, it is not clear what they are or how they change. There is also a wide range of tasks used to elicit oral language, and in this same vein, this could affect the outcome, as voice features would depend on the specific processes involved in the task.

Given these challenges, we believe the time is right to review the advances made in the field. We intend to discover whether these past 10 years have uncovered parameters related to those cognitive impairments, whether these parameters are useful for diagnosing AD and MCI, whether the method is reliable and straightforward, which tasks have been used the most, and how the related studies have evolved. The heterogeneity involved calls for consensus on this matter in order to advance.

The objective here is to conduct a systematic review that explores the issue of speech analysis in AD and MCI. We aim to critically evaluate the quality of the evidence on this subject, thereby proposing the following research questions:

1. What features characterize the speech of people with AD and MCI?
2. Is automatic speech analysis a reliable method for assessing AD and MCI?
3. Which is the most useful task used for eliciting oral language?

## Method

### Eligibility Criteria

Use was made solely of peer-reviewed journal articles applying speech signal processing techniques to the voices of people with AD or MCI. They were required to involve at least one AD or one MCI group, as well as a healthy control group consisting of older people. Articles that explored other neurodegenerative disorders but provided results for these groups were also included. They were required to record spoken utterances and analyze them by means of automatic speech analysis techniques. Studies that only contained manually extracted speech parameters were excluded. Only studies that aimed at either exploring voice characteristics or diagnosing AD or MCI were included. The outcomes consist of either descriptive voice parameters altered in people with MCI or AD with respect to a healthy older population or diagnostic accuracy to distinguish between these groups.

The exclusion criteria were as follows: (1) studies that did not use speech signal processing techniques; (2) studies without a group of AD and MCI; (3) studies in a language other than English or Spanish; (4) studies not published in a peer-reviewed journal; (5) studies that are narrative or systematic reviews; and (6) case studies.

### Method for Locating and Identifying Studies

The search was conducted in the electronic databases PubMed, CINAHL, and PsychINFO. The last search was conducted on 10 June 2020. The same terms were included in all the databases (speech features OR acoustic features OR voice OR speech analysis OR acoustic analysis OR speech signal OR spoken language OR speech production OR spontaneous speech OR connected speech OR speech acoustics OR automatic spontaneous speech analysis OR ASSA) AND (Alzheimer OR mild cognitive impairment).

The results were collected, and duplicates were removed. Two reviewers (IM and TEL) then independently reviewed the titles and abstracts of the studies retrieved. This phase required reading the full text several times, with the main reason being that the method for analyzing speech was not clearly described in the abstract. The interrater agreement was 0.991, κ = 0.992. Disagreements between the reviewers were resolved by discussion. Those articles that did not meet the inclusion criteria were removed. The PRISMA statement (Moher et al., 2009) was followed for conducting this review.

### Quality Assessment and Data Extraction

The methodological quality and risk of bias of the selected studies were assessed through two checklists: JBI critical appraisal checklist (Moola et al., 2020) for the analytical cross-sectional study, and the QUADAS-2 (Whiting et al., 2011) tool for the quality assessment of diagnostic accuracy studies.

Sample size and the method for eliciting speech were retrieved from all the articles. One of the aims of this review is to identify relevant features that characterize the voices of people with AD or MCI. We therefore looked for significant differences in the values of rhythmic and acoustic features between healthy controls and those groups. When the studies sought to classify the patients into AD or MCI according to the information provided by their speech signal, we selected the accuracy values whenever possible in order to compare the effectiveness of the different methods. If accuracy values were not provided, the most relevant value was selected. When more than one accuracy value was given, the lowest and highest are reported.

## Results

**Figure 1:**
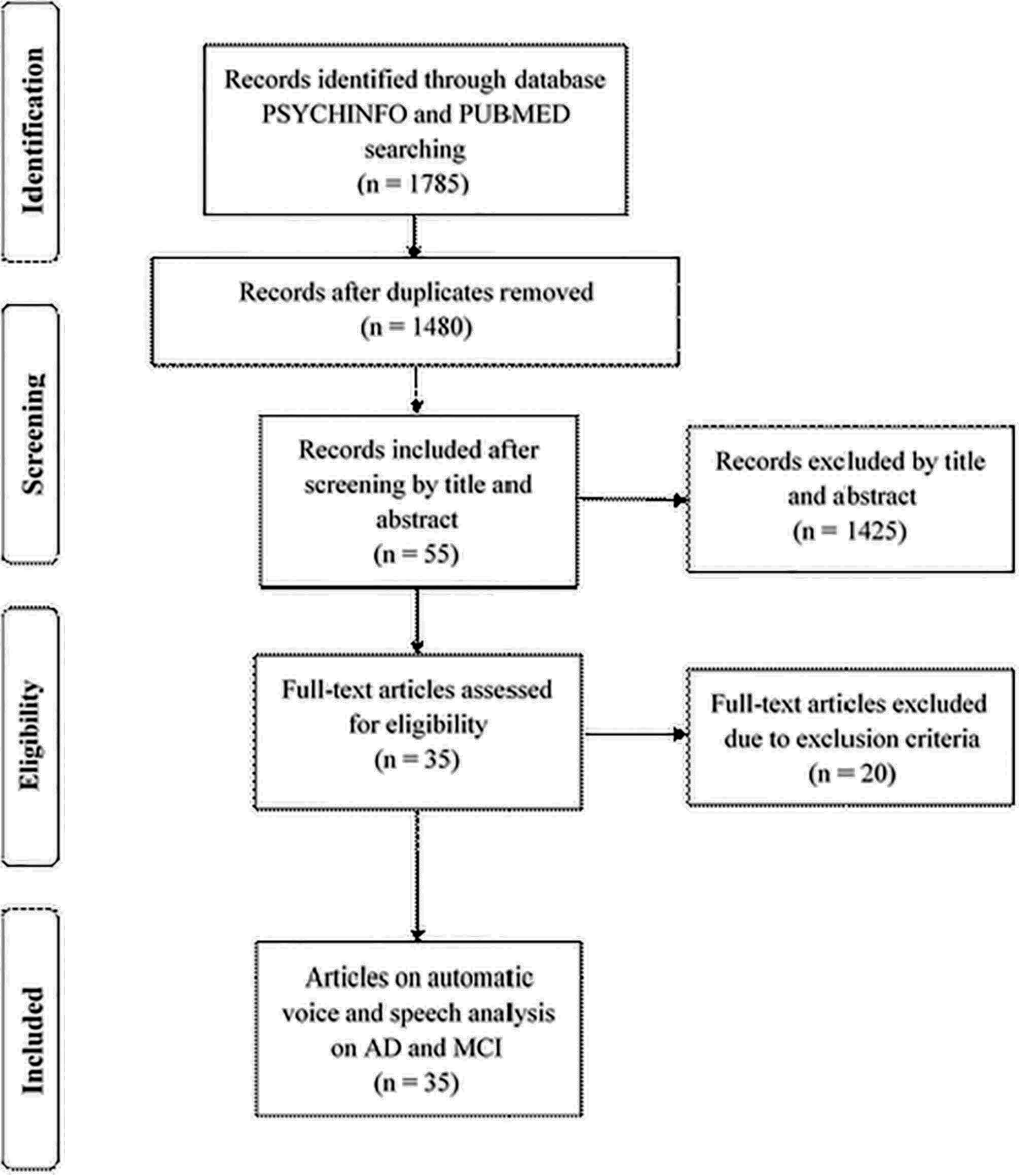
PRISMA flowchart of the process followed to select studies for the review.

The search process has been summarized in Figure 1 through a PRISMA flowchart. A total of 1,785 studies were retrieved after the search; 305 duplicates were removed. After screening by title and abstract, 55 studies were selected for a full-text review, and 20 of them were eliminated after the exclusion criteria were applied. This meant a total of 35 articles were finally included. The most common reason for exclusion was the lack of an automatic analysis of the speech signal, as many studies focused on discourse and content analysis. Another important reason was the use of this technique for pathologies other than AD or MCI, such as Parkinson or schizophrenia.

Among the studies selected, one examined solely AD patients, one examined solely MCI, 18 compared healthy control and AD groups, six compared control and MCI groups, and nine compared control, MCI, and AD groups. The methods used to elicit speech are diverse, and many studies use more than one. The most common are structured conversations, reading tasks, and standardized tasks, such as picture description (e.g., the “Cookie Theft” picture from the Boston test) or verbal fluency tasks. Other tasks used occasionally include recalling events or videos, and speaking about daily life. Further and more detailed information about sample sizes and the method for eliciting speech signals can be found in Tables 1, 2.

**Table 1:** Descriptive studies on voice and speech of Alzheimer's disease and mild cognitive impairment patients.

**References**	**Sample**	**Task**	**Main finding**
Hoffmann et al. (2010)	Control (15) and AD (30)	Spontaneous speech: explain why they are at the clinic and recount events and daily activities	Longer speech time and phonation time; higher voice breaks/hesitations >30 ms; lower speech rate and articulation rate
Horley et al. (2010)	Control (20) and AD (20)	Sentence repetition with a given emotional tone and reading	Differences in F0 (only when expressing surprise or happiness), F0 SD, and speech rate (when reading, but not when repeating).
Martínez-Sánchez et al. (2012)	Control (17) and AD (25)	Oral reading	F0, F0 SD, phonation time, proportion of pauses, % voiceless segments, voice breaks
Nasrolahzadeh et al. (2016a)	Control (30) and AD (30)	Telling personal stories and conversation	AD patients record fewer variations in speech signal
Nasrolahzadeh et al. (2016b)	Control (30) and AD (30)	Telling personal stories and conversation	Spontaneous speech signals of AD patients are less chaotic and non-linear than healthy subjects using higher-order spectra analysis.
Beltrami et al. (2018)	Control (48) and cognitively impaired (48: 16 aMCI, 16 mdMCi, and 16 eDem)	Describing a complex picture, a typical working day, and recalling the last dream remembered	Acoustic and rhythmic features can differentiate between multidomain MCI, early dementia, and control. Some acoustic features discriminated between control and aMCI. Differences in spectral centroid; standardized pause rate; transformed phonation rate; duration of speech segments; duration of silence segments
Meilán et al. (2012)	AD (21)	Reading	% of voiceless segments explains a significant portion of the variance in the overall scores obtained in the neuropsychological test of patients with AD
Meilán et al. (2018)	Control (102), MCI (38), and AD (42)	Reading	Semantic and phonetic verbal fluency tasks explain 30.1% of the variance of unvoiced percentage and 26.4 of the percentage of voice breaks.
De Looze et al. (2018)	Control (36), MCI (16), and AD (18)	Reading sentences of different length and syntax complexity	Changes in speech chunking and speech timing when reading cognitive demanding sentences may be markers of MCI and AD as a consequence of impairments in working memory and attention.
Qiao et al. (2020)	Control (24), MCI (20), and AD (20)	“Cookie theft” picture description task	The 7 parameters found correlate with the cognitive function. Stepwise regression showed that the maximum and average duration of silence segments, the percentage of the duration of silence, and the minimum duration of phrasal segments explain 47.8% of the MMSE score variation.
Meilán et al. (2020)	Non-degenerative MCI (73) and MCI preAD (13)	Reading task	Duration and phonation time, pause number, and several frequency and intensity features differentiate people with MCI that develop Alzheimer from those that do not.

**Table 2:** Predictive studies on the early diagnosis of Alzheimer's disease.

**References**	**Sample**	**Task**	**Parameters**	**Predictive value**	**Analysis method**
López-de-Ipiña et al. (2013a)	Control (20) and AD (20)	Telling stories and conversation	Combination of two feature sets: emotional speech analysis (acoustic, voice quality, and duration features) and emotional temperature (prosodic and paralinguistic features)	AD: 75.2–97.7%	Artificial neural networks
López-de-Ipiña et al. (2013b)	Control (20) and AD (20)	Telling stories and conversation	% voiced, % voiceless segments	AD: 83.7–93.79%	Machine learning
Martínez-Sánchez et al. (2013)	Control (35) and AD (35)	Reading task	Cut-off points for speech rate is 3.08 syllables per second, and for articulation, it is 4.27 syllables per second.	AD: 80%	ROC curve
Meilán et al. (2014)	Control (36) and AD (30)	Reading task	Percentage of voice breaks, number of periods of voice, number of voice breaks, and shimmer (apq3)	AD: 84.4%	Linear discriminant analysis
Khodabakhsh et al. (2015)	Control (27) and AD (27)	Conversation	Combination of 13 features (voice activity, articulation, and rate of speech-related features)	AD: 75.5–94.3%	Machine learning
Khodabakhsh and Demiroglu (2015)	Control (51) and AD (28)	Unstructured conversation	Silence ratio	AD: 78.5–83.5%	Machine learning
López-de-Ipiña et al. (2015a)	Control (20) and AD (20)	Telling stories and conversation	Combination of features	AD: 96.89%	Machine learning
López-de-Ipiña et al. (2015b)	Control (20) and AD (20)	Telling stories and conversation	Automatic selection of spontaneous speech features and of maximum, minimum, variance, standard deviation, median, and mode average for full signal and voiced signal	AD: 87.30–92.43%	Machine learning
Martínez-Sánchez et al. (2017)	Control (82) and AD (45)	Reading	Standard deviation of the duration of syllabic intervals	AD: 87%	ROC curve
Nasrolahzadeh et al. (2018)	Control (30) and AD (30)	Telling personal stories and conversation	Higher-order spectral analysis	AD: 94.18–97.71%	Machine learning
König et al. (2015)	Control (15), MCI (23), and AD (26)	Counting backward task, sentence repeating task, image description task, and verbal fluency task.	Combination of meaningful vocal features extracted from several tasks	MCI: 79%; AD: 87%; MCI vs. AD: 80%	Machine learning
López-de-Ipiña et al. (2018a)	Control (187) and MCI (38)	Categorical verbal fluency task (animals)	Several types of features are used to model both linear and non-linear disfluencies and speech. A total of 920 features are obtained. The best results are achieved with the 25-feature set.	MCI: 92–95%	Deep learning
López-de-Ipiña et al. (2018b)	Three different samples: VF task (187 control and 38 patients with MCI); picture description (12 control and 6 with AD); spontaneous language (50 control and 20 with AD)	Categorical fluency task (control vs. MCI)Picture description task and spontaneous speech (control vs. AD)	Most relevant features automatically extracted for every comparison.	MCI: 73%; AD: 89–95% (from spontaneous speech task)	Deep learning
Kato et al. (2018)	Control (91), MCI (91), and AD (91)	Answering a questionnaire on birthplace (T1), the name of his/her elementary school (T2), time orientation (Q2), and repeating 3-digit numbers backward (Q6)	Speech prosody-based cognitive impairment rating (SPCIR; 128 prosody features)	AD: 74.7–89.5% (from Q2); MCI: 70.9–76.4% (from Q6)	ROC curve
Themistocleous et al. (2018)	Control (30) and MCI (25)	Reading (vowels were segmented from sentences)	Vowel formants, F0, vowel duration	MCI: 75–83%	ROC curve
Toth et al. (2018)	Control (36) and MCI (48)	Immediate and delayed recall of a short film	Combination of features (duration, speech rate, articulation rate. and pause-related).	MCI: 75%	Machine learning
Fraser et al. (2016)	Control (97) and AD (167)	“Cookie theft” picture description task	Combination of 35 to 50 acoustic, semantic, and syntactic features.	AD: 78.72–81.92%	Machine learning
Fraser et al. (2019)	Control (29) and MCI (26)	Cookie theft picture description task and reading task	Speech features + eye movement features + language features	MCI: 41–83%	Machine learning
Gosztolya et al. (2019)	Control (25), MCI (25), and AD (25)	Immediate and delayed recall of a short film	Set 1: acoustic features (speech rate and the number and duration of silent and filled pauses) Set 2: acoustic features + linguistic features	Set 1: 74–82% Set 2: 80–86%	Machine learning
König et al. (2018)	SCI (56), MCI (44), VD (38), and AD (27)	Fluency, picture description, counting down, and free speech tasks	Different combination of features extracted for every comparison	SCI vs. AD = 92%, SCI vs. VD = 92%, SCI vs. MCI = 86%, and MCI vs. AD = 86%.	Machine learning
Martínez-Sánchez et al. (2018)	Control (98) and AD (47)	Reading	Age, minimum amplitude, maximum amplitude difference, mean and standard deviation of the NHR; asymmetry; standard deviation in the first formant; formant 3 bandwidth; standard deviation of the Acoustic Voice Quality Index; tone variability; Normalized Pairwise Variability Index	AD: 92.4%	Discriminant analysis
Al-Hameed et al. (2019)	Control with memory complaints (15), neurodegenerative disorders (15: AD 10, 2 aMCI, 2 frontotemporal dementia)	Conversation	Different sets of features (best with 9 features)	Neurodegenerative disorders: 81–92%	Machine learning
Chien et al. (2019)	Control (30) and AD (30)	Answers to neuropsychological tests	Feature sequence (a representation of various elements in speech)	AD: AUC 0.838	Machine learning
Nagumo et al. (2020)	Control (6343), MCI (1601), global cognitive impairment (367), MCI + GCI (468)	Vowel utterances, tongue twister, diadochokinetic rate, short sentences	Set of temporal and acoustic features.	MCI: AUC 0.61	Machine learning

Two main trends were identified within the selected studies: 11 of them compared the voices of the groups in order to find characteristic features, describe them, and explain the differences; 24 sought to classify the sample into their corresponding group using their voices, that is, by developing a tool for the early diagnosis of MCI and AD. The review was simplified by organizing the studies into two tables according to their aims. Table 1 contains the descriptive studies, and Table 2, the prescriptive ones.

### Descriptive Studies on the Voice and Speech of AD and MCI Patients

The first study on the matter was published in 2010 by Hoffmann et al. (2010). They used PRAAT software (Boersma and Weenink, 2007) for automatically analyzing spontaneous speech signal samples from a healthy older control group and from an AD group. This study found several features affected by the disease, such as higher voice breaks, longer speech time and phonation time, and lower speech and articulation rate. That same year, another study by Horley et al. explored emotional prosody in AD patients, finding differences in F0 when expressing surprise or happiness, as compared with a control group. AD patients had an impaired prosody expression when trying to imitate emotional speech. These findings were replicated by other studies that besides changes in voice breaks and F0 found differences in pauses and voiceless segments (Martínez-Sánchez et al., 2012) and fewer variations in speech signal (Nasrolahzadeh et al., 2016a,b).

Regarding people with MCI, the efforts have mainly focused on detection, as will be noted in the next section. Nevertheless, Beltrami et al. (2018) have found features that characterize different types of MCI, with differences between amnestic MCI and multidomain MCI in rhythmic features such as the pairwise variability index, and especially in acoustic features such as the duration of silence and speech segments, phonation rate, pause rate, and spectral centroid. In a recent study, Meilán et al. (2020) have sought to find two different profiles within people with MCI and hypothesized that several features differentiate between people that will develop AD and people that will not deteriorate further.

This search uncovered four articles that set out to explain changes in terms of cognitive processes. Two of them aimed to explore whether a series of neuropsychological variables of lexical access could predict changes in speech features. Meilán et al. (2012) have found that a phonological verbal fluency task explains 46% of the variance in the voiceless segments. In another study, the same authors (Meilán et al., 2018) have reported that semantic and verbal fluency tasks could account for 30.1% of the variance in unvoiced percentage and 26.4% of the percentage in voice breaks. De Looze et al. (2018) have used sentences that differ in length and syntactic complexity, finding that their speech deficits are related to working memory and attention deficits in the case of people with MCI, and to language deficits in AD patients. Finally, the remaining study by Qiao et al. (2020) has found that seven features explain 47.8% of the variation in the MMSE score.

### Predictive Studies on the Early Diagnosis of AD and MCI

Three years after the publication of the first article on the automatic speech analysis of AD patients, the first three articles on classifying healthy controls and people with AD based on their speech signal were published almost simultaneously. López-de-Ipiña et al. (2013a) analyzed a spontaneous speech task with an accuracy of 97.7% using two sets of features that combined acoustic, voice quality, duration, prosodic, and paralinguistic features. The same team recorded 93.79% accuracy by using just two features: percentage of voiced and voiceless segments (López-de-Ipiña et al., 2013b). Martínez-Sánchez et al. (2013) used an oral reading task and obtained 80% accuracy through speech rate and articulation rate. That same group (Meilán et al., 2014) raised that value to 84.4% by using four features. After this start, different approaches would subsequently be developed. Khodabakhsh and Demiroglu (2015) and Khodabakhsh et al. (2015) recorded values ranging from 83.5% using just the silence ratio to 94% combining 13 features. López-de-Ipiña et al. (2015a,b) assayed different methods and combinations of features, obtaining 92–97% accuracy. With just one feature, namely, the standard deviation of the duration of syllabic intervals, Martínez-Sánchez et al. (2017) classified AD patients with 87% accuracy. Nasrolahzadeh et al. (2018) achieved 97.71% accuracy using higher-order spectral analysis with a spontaneous speech task. Finally, Chien et al. (2019) devised a new method of representing elements in speech, with an AUC of 0.838.

In 2015, the first study using automatic speech analysis to identify MCI was published. König et al. (2015) compared the voices of healthy older adults, people with MCI and AD patients. They extracted features that showed significant differences in several tasks and obtained the best combination through machine-learning methods. This gave an accuracy of 79% for MCI and 87% for AD. Furthermore, they could differentiate people with MCI from those with AD with 80% accuracy. López-de-Ipiña et al. (2018a,b) also used combinations of features with 73–95% accuracy for MCI. By combining prosodic features produced while repeating numbers backwards, Kato et al. (2018) classified people with MCI with 76.4% accuracy. Themistocleous et al. (2018) focused on formants produced while reading, thus achieving an accuracy value of 83%. The last study of this kind found in the search was by Toth et al. (2018), who used features related to duration, speech rate, articulation rate, and pauses to obtain a 78.8% F1-score through machine-learning methods. Al-Hameed et al. (2019) gathered a group with several neurodegenerative disorders including AD and aMCI and properly classified them with 92% accuracy. The study with the largest sample to date has been published recently (Nagumo et al., 2020), and it is also the most disappointing in terms of results. With more than 8,000 participants, they only managed an AUC of 0.61, although as the authors themselves indicate this could be due to an unreliable classification of the sample. However, they defend speech analysis as a good measure of the severity of the impairment.

Three studies have combined automatic speech analysis with other techniques in search of better results. For instance, Fraser et al. (2016) have classified healthy older people and AD patients with 81.92% accuracy by adding sundry semantic and syntactic features to the acoustic ones. They have subsequently used speech recordings, eye movement tracking, and language features, achieving 83% accuracy differentiating between healthy control and MCI groups (Fraser et al., 2019). A study by Gosztolya et al. (2019) has improved the classification of control, MCI, and AD groups, from 74–82% accuracy when using just acoustic features to 80–86% when adding linguistic features.

Finally, there is another significant type of studies among those that aim to predict AD and MCI. As of 2018, the knowledge accumulated has allowed going a step further with the emergence of studies programming automatic speech analysis in devices or applications that clinicians could use as a diagnostic tool. This search has found two approaches to the matter: the first of them, by König et al. (2018), uses a mobile application to discriminate between people with subjective cognitive impairment (SCI), MCI, vascular dementia (VD), and AD, with an accuracy ranging from 86 to 92%. At the same time, Martínez-Sánchez et al. (2018) have presented a device that contains an algorithm that uses nine acoustic features to predict AD with an accuracy of 92.4%.

### Methodological Quality According to JBI and QUADAS-2 Criteria

**Figure 2:**
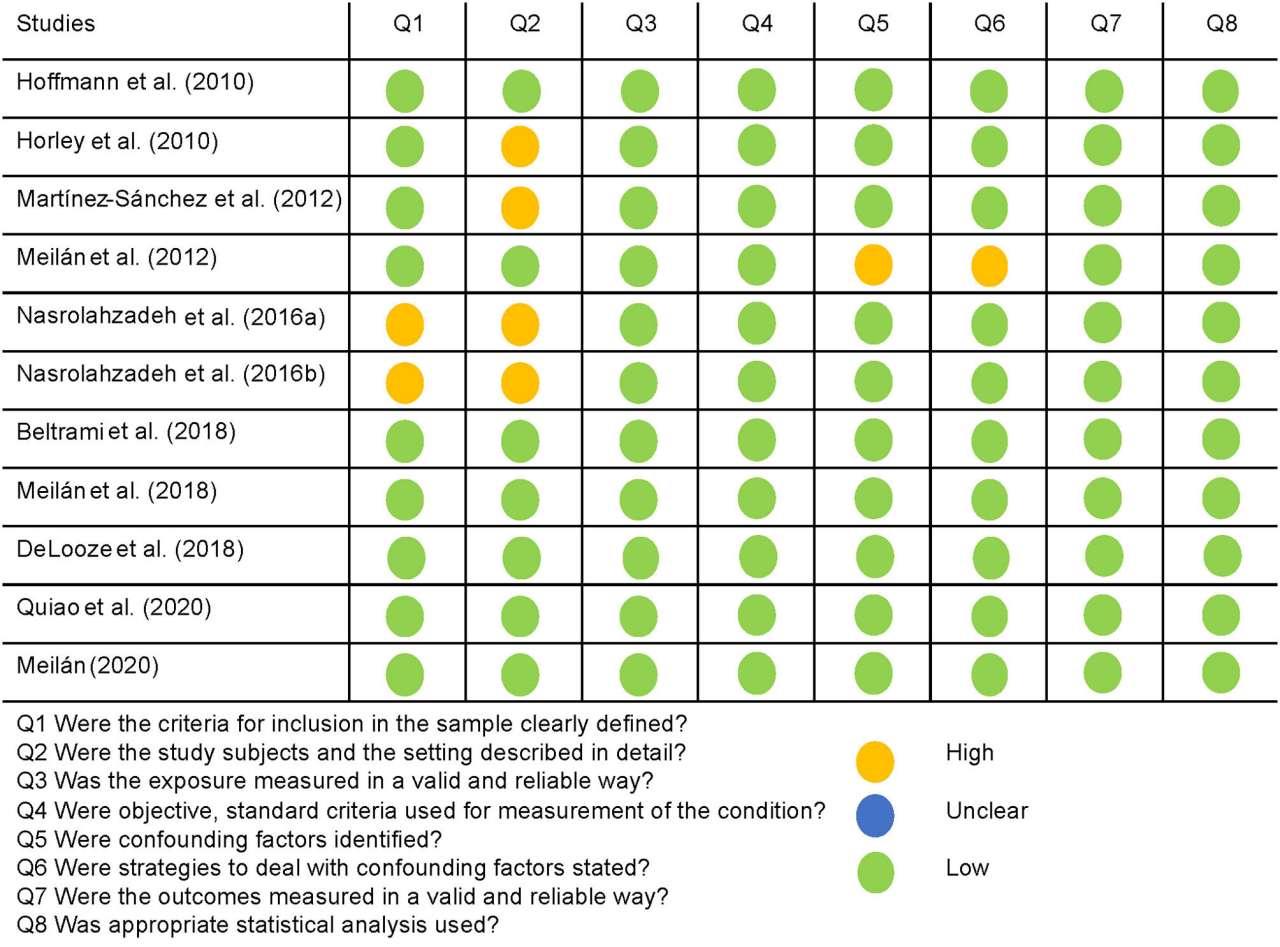
Quality assessment of the descriptive studies using the JBI appraisal checklist, and their rating is a high, low, or unclear risk of bias for each question.

**Figure 3:**
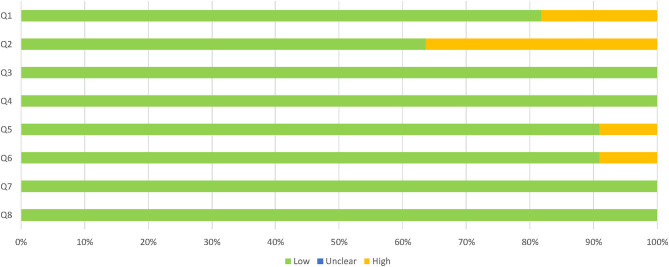
Proportion of descriptive studies with a low, high, or unclear risk of bias.

Descriptive studies recorded good quality overall, as assessed by the JBI critical appraisal checklist for analytical cross-sectional studies. The specific assessment of each dimension of the checklist as having a high, low or unclear risk of bias for every study can be seen in Figure 2, with a summary of the overall results in Figure 3. More than half of the studies (54.5%) suitably addressed all the questions. The most common concerns arose from the definition of the sample: two studies lacked inclusion criteria, and four of them did not clearly or sufficiently describe the sample in objective terms. However, the assignment to the groups was carried out following NINCS-ADRA or similar criteria. Only one study did not deal with potentially confounding factors; the rest of them used either matching paired participants or adjustment in data analysis.

**Figure 4:**
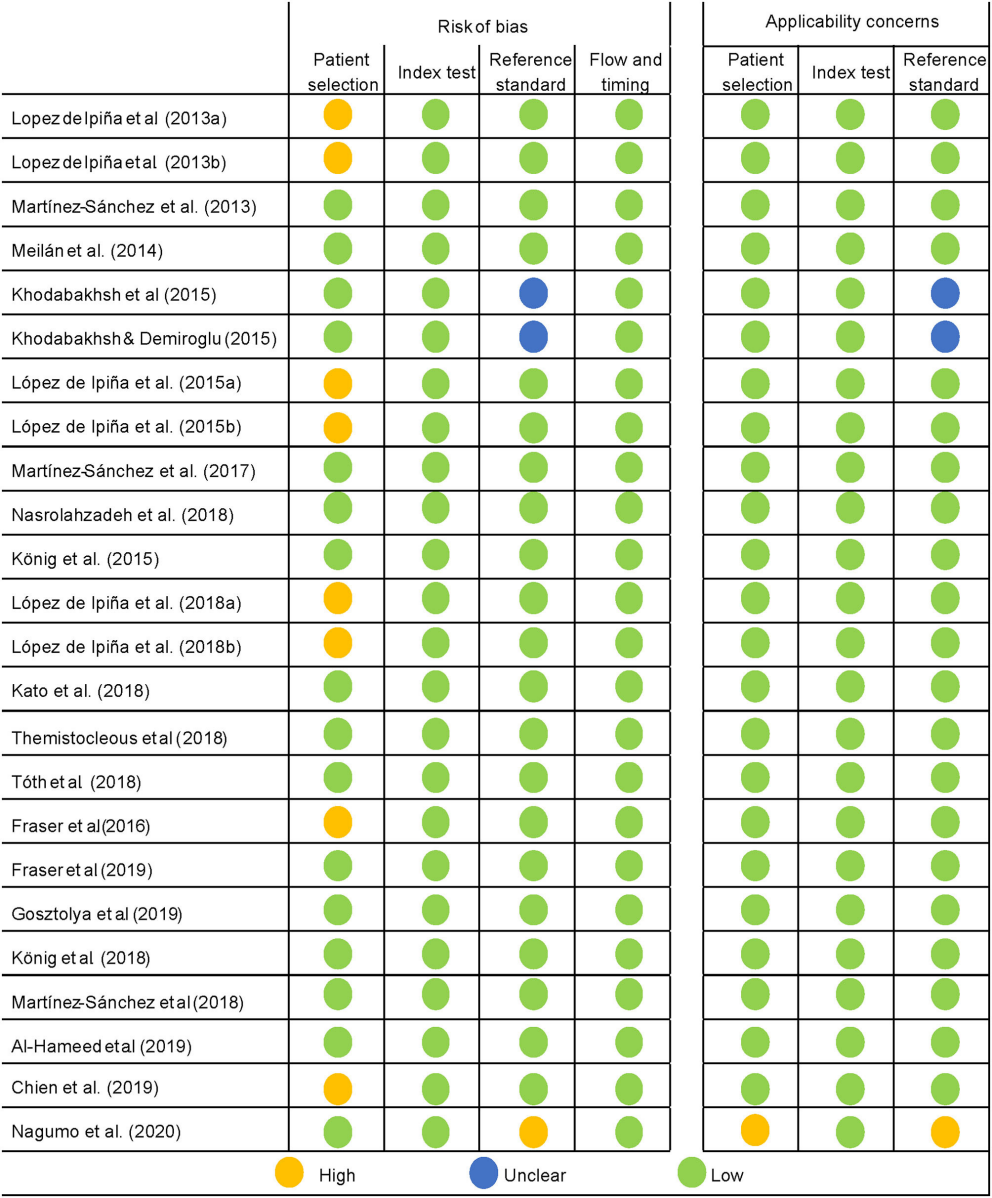
Quality assessment of the predictive studies using the QUADAS-2 checklist and their rating as a high, low, or unclear risk of bias for each domain and their applicability concerns.

**Figure 5:**
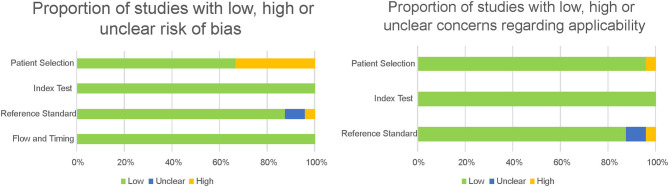
Proportion of predictive studies with a low, high, or unclear risk of bias.

The QUADAS-2 tool was used for the quality assessment of predictive studies. The quality of the studies is either acceptable or high (see Figures 4, 5). The main concerns were in the patient selection domain, as in the descriptive studies. Several of the studies assessed did not use a consecutive sequence nor a random selection of the sample, but rather selected a sample of patients and then a matching sample for the control group. Although not a major flaw, it does raise some concerns, and it is a possible source of bias. Most studies received a low bias rating for the reference standard as appropriate tests were used. In the cases marked as unclear, not enough information was provided on the tests used. When the rating was a high risk of bias, the tests they used were not sufficient to assign the diagnostic category. Most studies received a low risk of bias inapplicability concern domains, as patient selection, index test, and reference standard matched the aims and questions of the assessed studies.

In sum, it seems safe to say that there is robust evidence of cognitive changes in the voices of older people and their pertinence for detecting MCI and AD, when measured by the automatic analysis of the speech signal. The concerns raised regarding the risk of bias in the studies were more commonly found in the first few years, with more recent studies recording greater accuracy, which ultimately means this is a mature and soundly grounded field.

## Discussion

The study of the automatic speech analysis of the voices of people with AD and MCI has being evolving over the years.

In the first period, starting in 2010, the efforts focused on the extraction of features that defined the voices of patients with AD. These studies would soon lead to the first attempts, as early as 2013, at identifying AD by means of speech signal analysis. The success achieved by these last studies caused a change in the focus of study. In 2015, the first studies on MCI appeared, still coexisting with research designed to refine the method for AD and achieve better results. Although less precise, MCI diagnosis was promising, and more complex methods would be applied in the quest for higher accuracy. In 2018, there was a major step forward: the first attempts were made to extrapolate lab results to clinical practice. The inclusion of the procedure on devices and applications with no loss of accuracy was a major achievement because it is quick, non-invasive, reliable, and cheap. That is a reflection of the advances in the matter, as it means it is sufficiently developed to make the leap from the laboratory to daily clinical practice.

As has been made clear, automatic speech analysis can detect subtle changes in the voices of people suffering from neurodegenerative processes such as MCI and AD. A summary of these changes can be found in Table 3, which details the main features of studies that show changes for both groups in comparison with healthy older people. This table shows which parameter changes are associated with MCI or more advanced stages of dementia according to the studies reviewed. In the early stages of AD, patients show changes in several temporal and acoustic parameters, such as a decrease in speech and articulation ratio and an increase in the number of pauses (López-de-Ipiña et al., 2013a; Toth et al., 2018). There is a greater continuity of periodic and harmonic segments for healthy older adults than for those with MCI, which is even less in people with AD (König et al., 2015). Additionally, changes in spectrum features, such as fundamental frequency and formants (Themistocleous et al., 2018), can be found in people with MCI and AD. In the speech of patients with AD, the aforementioned symptoms worsen over the course of the disease, resulting in longer phonation time (Hoffmann et al., 2010; Martínez-Sánchez et al., 2012; König et al., 2015), an increase in the number and proportion of pauses (Martínez-Sánchez et al., 2012, 2013), and lower speech and articulation rates (Hoffmann et al., 2010; Horley et al., 2010; Martínez-Sánchez et al., 2013; König et al., 2015). Other impairments become more severe, presenting more voice breaks (Hoffmann et al., 2010; Meilán et al., 2014; König et al., 2015), a higher percentage of voiceless segments (Meilán et al., 2012; López-de-Ipiña et al., 2013b), wider variability in autocorrelation values (Meilán et al., 2014), bigger changes in spectral features such as the spectral centroid (Fraser et al., 2016), fundamental frequency, and a distortion in formants—especially in F3—(Meilán et al., 2014; Khodabakhsh and Demiroglu, 2015), and a lower noise-to-harmonics ratio (Meilán et al., 2014). It is difficult to know from these studies whether some parameters carry more weight than others in diagnosis. Some studies have explored a single parameter—speech rate (80%), silence ratio (78%), standard deviation of the duration of syllabic intervals (87%)—recording worse, albeit still good, results compared with studies using large sets of features. The most common studies use combinations of several parameters, improving accuracy to above 90%; however, none of them explores these parameters' individual roles. Whether explored individually or in sets, it seems that the most frequently used parameters are the temporal and prosodic ones. It should be noted that several studies do not report the features found and refer to sets or groups of parameters, usually obtained after using machine-learning methods on large sets of features in order to find the most powerful one for distinguishing between the diagnostic entities. Although it serves the purpose of simplifying the results of articles that sometimes refer to dozens of parameters, they hinder the consensus we are trying to reach.

**Table 3:** Review of studies on the distinctive speech markers in older people.

	**Mild cognitive impairment**	**People with Alzheimer's disease**
**Temporal parameters**		
Speech time and phonation time	Longer in MCI (Toth et al., 2018; Gosztolya et al., 2019).	Longer in AD than in healthy control (Hoffmann et al., 2010; König et al., 2015).
Number and proportion of pauses	Increase in the length of silent pauses (voiceless), producing a lower speech rate (Toth et al., 2018).	Increased number and proportion of pauses in AD (Martínez-Sánchez et al., 2012, 2013; López-de-Ipiña et al., 2013b).
Voice breaks/hesitations >30 ms		Higher in AD (Hoffmann et al., 2010; Martínez-Sánchez et al., 2012; Meilán et al., 2014; König et al., 2015).
% voiceless segments		Higher in AD. It explains a significant portion of the variance in the overall scores obtained in the neuropsychological testing of patients with AD (Meilán et al., 2012).
Prosodic rate: decrease in speech rate with hesitations, as well as in articulatory rate without hesitations (fewer phonemes per second).	Presence of stammers and articulatory disfluencies that interrupt speech; longer hesitations (López-de-Ipiña et al., 2013b; Toth et al., 2018).	Lower speech rate and articulation rate (Hoffmann et al., 2010; Horley et al., 2010; Martínez-Sánchez et al., 2013; König et al., 2015).
Affective prosody		Impairments in affective prosody expression in the AD group when expressing surprise or happiness, but not sadness (Horley et al., 2010).
**Phonological planning**		
Spectrum features		Changes in spectrum features such as special centroid or mel-frequency cepstral coefficients (MFCCs), the spectral energy, flux, variance, skewness, kurtosis, and slope (Fraser et al., 2016; Al-Hameed et al., 2019).
Fundamental frequency	Altered (Themistocleous et al., 2018)	Lower mean of the fundamental frequency and standard deviation causing a “flat” speech prosody (Horley et al., 2010; Martínez-Sánchez et al., 2012).
Autocorrelation: fluctuation of values of autocorrelation of the fundamental frequency in a specific period		Wider variability (Meilán et al., 2014).
Phonological planning: formants	Impairments in formant features (Themistocleous et al., 2018)	Distortion in the parameters F2 and F3 (Meilán et al., 2014). Slower changes between formants (Khodabakhsh and Demiroglu, 2015)
Syllabic variability		Altered mean duration of syllables (Martínez-Sánchez et al., 2013). Greater standard deviation of the duration of syllabic intervals (mean variability of the duration of syllables) (Martínez-Sánchez et al., 2017).
**Quality of voice measures**		
(NHR): noise/harmonics		Lower ratio in AD than in people with NPS (Meilán et al., 2014).
Continuity of harmonic segments		Lower in AD (König et al., 2015).
**Amplitude parameters**		
Shimmer		Decrease amplitude perturbation quotient of sound between 3 and 11 vocal pulses (Shimmer_dB apq3; Shimmer_dB apq11) (Meilán et al., 2014).

Automatic speech analysis as a tool for diagnosing MCI and AD is the aspect that has informed most publications. The accuracy values for AD range from 80 to 97%, which is a significant difference. In order to facilitate the comparison with other tools, we will consider only those values from the studies involving applications or devices, given that they are the most similar to clinical tools. In these cases, the accuracy for AD is about 92%. For MCI, most values range between 73 and 86% or 82 and 86% in the studies with applications. Studies that use biomarkers such as the volume of hippocampus measured with MRI (Chupin et al., 2009) or amyloid PET tracers (Morris et al., 2016) record accuracies of over 90% months or even years before the definitive diagnosis. Although the results of biomarker studies are better because they can identify the disease before the symptoms arise, they are extremely expensive, time consuming, and only highly specialized professionals can conduct them. Therefore, the analysis of a speech signal is far cheaper and straightforward, and it can be conducted on everyday devices such as mobile phones, making it a valuable screening test.

Regarding the method used for eliciting oral language, it is a challenge to establish whether there is a more effective task. Most studies explore spontaneous speech, and only a few analyze reading tasks. The results are fairly similar, and both tasks reveal the same altered features in AD, such as longer speech time, lower speech rate, and an increased number of pauses. In the predictive studies on AD, the results are variable, and most of them range from 80 to 95% accuracy regardless of the type of task. In the case of MCI, the highest accuracy (95%) is achieved through a semantic verbal fluency task (López-de-Ipiña et al., 2018a), which is in marked contrast to the range normally found using other linguistic tasks (80–85%). However, it is not possible to affirm that semantic verbal fluency tasks are the most effective ones, as some of those studies also used acoustic features extracted during the execution of the task. More cognitively demanding tasks would be expected to cause greater differences, but that does not always seem to be the case. In turn, the use of different materials can be useful when exploring cognitive processes. Speech analysis serves as a direct measure of the execution of certain processes that will depend on the type of language. For example, reading implies a characteristic prosody (Dowhower, 1991) and differences are expected with respect to the prosody of spontaneous speech. Other factors may influence this. A clear example is the manipulation of the material in the study by De Looze et al. (2018), in which increasing the complexity of the sentences to be read by people with AD increases the number of pauses. Therefore, the best approach at present seems to be to conduct several tasks and select different features from each one in order to combine them in a more powerful final algorithm.

Considering the evolution this field has experienced over the last decade, it is likely that the next steps will be taken in the direction of differential diagnoses for neurodegenerative pathologies. Moreover, in the near future new studies are expected to try to predict AD and the conversion from MCI to AD, even before any symptoms arise, as the tendency is to involve patients in earlier stages. In this sense, identifying certain types of MCI (amnestic, multidomain) would be of major significance, as they constitute a risk factor for different kinds of dementia. Longitudinal studies should therefore be a priority. Another expected orientation is to explain the changes in the voice in cognitive terms, a topic on which there was initially little progress, and which has been gaining strength since 2018. These changes in the voice are explained mainly by lexical-semantic impairments and the deterioration of executive processes and attention. It is noteworthy that there are studies that use the manual extraction of parameters such as pauses (Pistono et al., 2019), which they also attribute to lexical-semantic processes, and are understood as compensatory mechanisms to improve lexical selection and memory recall. Finally, the clinical use of this tool is another major step. As we have discussed, automatic voice analysis is a fast, cheap, and reliable screening method. We have already pointed out that there are several initiatives that use small devices (Martínez-Sánchez et al., 2018) and mobile applications (König et al., 2018), and not only those included in the review but also others not yet published in peer-reviewed journals, such as the use of a tablet posited by Hall et al. (2019). This is an important goal, as these formats allow us to convey the tool to any professional with a smartphone.

## Conclusion

There are other recent reviews that deal with the subject of spontaneous speech and dementias. However, they focus on other neurodegenerative processes (Boschi et al., 2017), conduct a general analysis of speech elicited by pictures in AD (Mueller et al., 2018; Slegers et al., 2018), or make a comprehensive but unsystematic review (Szatloczki et al., 2015). Shortly before the submission of this paper, a review was published by Pulido et al. (2020) in which they make an excellent contribution by providing an in-depth analysis of the multiple methods and databases commonly used in the field. Nevertheless, we believe that the one we present provides added value by contributing the risk of bias analysis, as it is essential to assess the validity of the evidence found. In any case, the proliferation of these studies shows the growing interest in language impairments in AD and MCI.

This review confirms that the analysis of speech signal among people with AD and MCI is a useful tool for detecting subtle changes in their language. There are indeed rhythmic and acoustic features that characterize the voices of these patients and can be used with great success to diagnose their condition. It constitutes an efficient, cheap, and easy-to-use tool that may facilitate the screening of dementias.

## Data Availability Statement

The original contributions presented in the study are included in the article/supplementary material, further inquiries can be directed to the corresponding author.

## Author Contributions

IM-N and JM conceived and planned the paper. IM-N conducted the search. IM-N and TL analyzed the results. IM-N wrote the manuscript with support of all authors. JM and FM-S provided critical feedback. All authors contributed to the article and approved the submitted version.

## Conflict of Interest

The authors declare that the research was conducted in the absence of any commercial or financial relationships that could be construed as a potential conflict of interest.
